# Efficacy of Postoperative FOLFOX *Versus* XELOX Chemotherapy for Gastric Cancer and Prognostic Value of Platelet–Lymphocyte Ratio in Patients Receiving XELOX

**DOI:** 10.3389/fonc.2020.584772

**Published:** 2020-12-23

**Authors:** Xin Yin, Tianyi Fang, Yimin Wang, Chunfeng Li, Yufei Wang, Daoxu Zhang, Yingwei Xue

**Affiliations:** Department of Gastroenterological Surgery, Harbin Medical University Cancer Hospital, Harbin Medical University, Harbin, China

**Keywords:** gastric cancer, postoperative chemotherapy, oxaliplatin capecitabine, platelet–lymphocyte ratio, systemic immune inflammation, prognosis, nomogram

## Abstract

**Background:**

Surgery combined with postoperative chemotherapy is an effective method for treating patients with gastric cancer (GC) in Asia. The important roles of systemic inflammatory response in chemotherapy have been gradually verified. The purpose of this study was to assess the difference in clinical effectiveness of FOLFOX (oxaliplatin + leucovorin + 5-fluorouracil) and XELOX (oxaliplatin + capecitabine), and the prognostic value of postoperative platelet–lymphocyte ratio (PLR) in the XELOX group.

**Methods:**

Patients who received radical gastrectomy combined with postoperative chemotherapy between 2004 and 2014 were consecutively selected into the FOLFOX and XELOX groups. Group bias was reduced through propensity score matching, which resulted in 278 patients in each group. Cut-off values of systemic immune inflammation (SII) score and PLR were obtained by receiver operating characteristic curve. Kaplan–Meier and Log-rank tests were used to analyze overall survival. The chi-square test was used to analyze the association between clinical characteristics and inflammatory indexes. Univariate and multivariate analyses based on Cox regression analysis showed independent risk factors for prognosis. The nomogram was made by R studio.

**Results:**

Patients receiving XELOX postoperative chemotherapy had better survival than those receiving FOLFOX (*P <* 0.001), especially for stage III GC (*P* = 0.002). Preoperative SII was an independent risk factor for prognosis in the FOLFOX group, and PLR of the second postoperative chemotherapy regimen in the XELOX group, combined with tumor size and pTNM stage, could construct a nomogram for evaluating recurrence and prognosis.

**Conclusion:**

XELOX is better than FOLFOX for treatment of GC in Chinese patients, and a nomogram constructed by PLR, tumor size and pTNM stage can predict recurrence and prognosis.

## Introduction

Gastric cancer (GC) is the third most common cause of cancer mortality worldwide, and causes 723,000 deaths each year according to the International Agency for Research on Cancer (IARC) in 2012. With the increasing awareness of cancer prevention and treatment worldwide, the incidence of GC has been declining in some developed countries, but more than 70% of new cases come from developing countries, and 42.6% are from China (1, 2), which suggests that GC is still a major threat to human health. At present, radical gastrectomy remains the curative treatment for GC globally, and postoperative chemotherapy has been a standard component of the treatment in Asia (3). In the selection of postoperative chemotherapy regimens, FOLFOX (oxaliplatin + leucovorin + 5-fluorouracil) and XELOX (oxaliplatin + capecitabine) are common regimens that have been widely used clinically after decades of clinical research (4). However, few studies have directly compared their efficacies, and choosing a suitable chemotherapy regimen is still a topic of discussion among clinicians.

The representative CLASSIC trial demonstrated a survival benefit for XELOX in patients with stages II–III GC (5, 6). Louvet et al. (7) showed that FOLFOX has good clinical efficacy in advanced GC. However, the high degree of heterogeneity of GC affects the clinical efficacy of different chemotherapy regimens. Baumgartner et al. (8) found that for palliative treatment of gastroesophageal cancer, XELOX was better than FOLFOX. Currently, there is still a lack of data demonstrating the feasibility of which chemotherapy regimen is more suitable for Chinese patients after radical gastrectomy.

The important role of tumor immunity in tumor progression is widely recognized. Immunological factors, especially in the peripheral blood, are considered to be potential biomarkers for prognosis and even early diagnosis of cancer and to guide postoperative chemotherapy. In 2014, Galon first proposed combination of immune response in the tumor microenvironment with traditional pathological staging based on tumor burden, presence of cancer cells in regional lymph nodes, and metastases to construct TNM-Immune (TNM-I)). In 2018, The *Lancet* first published the application of immune score for predicting postoperative chemotherapy sensitivity of patients with colon cancer. Pathological immunity evaluation may provide reliable information on tumor prognosis (9, 10). For early diagnosis of GC, Fang et al. (11) demonstrated that neutrophil–lymphocyte ratio (NLR) and platelet–lymphocyte ratio (PLR) were significantly better than traditional tumor markers. Lee et al. (12) found that patients with NLR ≥3 had worse survival after postoperative FOLFOX chemotherapy. Similarly, the dynamic changes in circulating immune cells also can be used to evaluate the effect of adjuvant therapy. Wang et al. (13) found that timing of neutropenia may be a potential prognostic biomarker, and Yumiko et al. (14) found that patients whose NLR increases by two at 60 days after surgery might not be suitable for nivolumab monotherapy. The systemic inflammatory response has an important role in influencing tumor progression and evaluating prognosis. Therefore, it is important to develop a simple and convenient inflammation index as a part of cancer classification and a prognostic tool for GC patients after radical gastrectomy and postoperative chemotherapy.

## Materials and Methods

### Patients

We consecutively selected 652 patients with GC in the Department of Gastrointestinal Surgery, Harbin Medical University Cancer Hospital between 2004 and 2014. The diagnosis was based on paraffin sections and confirmed by experienced pathologists after surgery. All patients underwent radical gastrectomy with R0 resection. During hospitalization of the patient, routine preoperative examinations were performed, including stomach computed tomography/magnetic resonance imaging, chest radiography, abdominal ultrasonography, electrocardiography, and hematological examinations and some patients underwent positron emission tomography-computed tomography (PET–CT) as needed.

Exclusion criteria were (1): preoperative chemotherapy; (2) antiplatelet agent therapy within 3 months before surgery; (3) intravascular coagulation; (4) active bleeding; (5) concurrent abdominal and other systemic infections or severe cardiovascular disease; (6) patients with blood malignancies, including multiple myeloma and (7) patients who did not complete postoperative chemotherapy as required.

According to the postoperative chemotherapy regimens, patients were divided into the FOLFOX and XELOX groups. Clinicopathological data were saved in the Gastric Cancer Information Management System v1.2 of Harbin Medical University Cancer Hospital (Copyright No.2013SR087424, *http:www.sgihmu.com*): sex, age, tumor size, Borrmann type, tumor location, hematological examination, histological type, vascular infiltration, and lymph node dissection. The pTNM stage and histology type were according to the 8th edition American Joint Committee on Cancer (AJCC). All patients were re-examined through checking radiological examination (ultrasound, CT and gastroscopy) and tumor markers every 6 months, and PET–CT was performed as needed.

### Inflammatory Index

Blood samples were collected from patients in fasting condition 1 week before surgery. For patients undergoing XELOX chemotherapy after surgery, blood samples were collected from patients in fasting condition in the first day for each time of postoperative chemotherapy. Blood (2 ml) from the cubital vein was collected and sent to the hematology laboratory where the serum was separated. For inflammation index, systemic immune inflammation (SII) score = N × P/L, neutrophil–lymphocyte ratio (NLR) = N/L, platelet–lymphocyte ratio (PLR) = P/L (N = neutrophil count, L = lymphocyte count, and P = platelet count)

### Chemotherapy

FOLFOX regimen: Day 1, oxaliplatin (85 mg/m^2^) in 500 ml normal saline or glucose, by intravenous infusion for 2 h. On Days 1 and 2, leucovorin (20 mg/m^2^), by intravenous bolus, for 10 min; after the bolus, 5-FU (400 mg/m^2^), by rapid intravenous bolus, and then 5-FU (600 mg/m^2^), by continuous intravenous infusion for 22 h. XELOX regimen: oxaliplatin, 150 mg/m^2^, by intravenous infusion, on Day 1 of every 3 weeks, and capecitabine (Xeloda), 1,000 mg/m^2^, orally, twice daily from Day 1 to Day 14 every 3 weeks, and at the same time, myocardial nutrition, liver protection, acid inhibition, and anti-vomiting therapies were given (4). FOLFOX regimen was performed at least six times and XELOX regimen was performed at least 8 times. All included patients received adequate chemotherapy without treatment discontinuation and dose reductions.

### Toxic and Adverse Effects

The main toxic and adverse effects of the two groups patients were bone marrow suppression and gastrointestinal reactions (including nausea, vomiting, and loss of appetite), as well as fatigue, oral mucositis, hand and foot syndrome, peripheral neurotoxicity, and liver and kidney damage. However, the toxic and adverse effects were grades I–III, with no grade IV, and were alleviated by symptomatic treatment (4).

### Statistical Analysis

To minimize the influence of confounding factors on selection bias, propensity score matching (PSM) was performed. The propensity scores were elicited from matched patients in 1:1 ratio with greedy matching algorithms without replacement. All clinical and pathological characteristics included sex, age, tumor size, Borrmann type, tumor location, lymph node dissection, histological type and vascular infiltration. Standardized differences for all characteristics before and after PSM were evaluated by chi-square test. If there is one Clinicopathological feature with a value of *P >*0.05, it was considered that there was a statistically significant selection bias between two groups. And there was no statistically significant selection bias existed when all characteristics had value of *P<*0.05.

Overall survival (OS) was determined, which was calculated as the time from surgery to death from any cause. Disease-free survival (DFS) was calculated as the time from surgery to recurrence in various forms. If patients were alive at last follow-up, they were censored. The 5-year OS in each group was compared. Log-rank test and Kaplan–Meier method were used to analyze the survival curves. The survival time was shown as median ± standard deviation.

The diagnostic significance of inflammatory indexes, including NLR, PLR and SII, for patients with GC was calculated and compared according to receiver operating characteristic (ROC) curve analysis. The area under the curve (AUC) was calculated, and the optimal cut-off value was analyzed by the Youden index, which was calculated by the sensitivity − (1 − specificity). The maximum value of the index was the optimal threshold. The dynamic changes of inflammation index and circulating immune cells was tested by non-parametric rank sum test (Mann−Whitney U Test). If *P <*0.05, it was considered that the change between the two measurements had significant statistical difference; if *P >*0.05, it was considered that there was no statistical difference. The chi-square test also was used to analyze the association between inflammatory index and clinicopathological features. *P <*0.05 was considered there was statistically significant association; *P >*0.05 was considered there was no statistically significant association.

Univariate and multivariate analyses based on Cox regression were used to analyze the independent risk factors for prognosis and recurrence, respectively. The indicators included clinicopathological features and the immune biomarkers with the largest AUC area calculated by ROC curve (SII was analyzed in FOLFOX group, and PLR was analyzed in XELOX group). Variables with a value of *P <*0.05 in the univariate analysis were subsequently included in a multivariate analysis, variables with a value of *P <*0.05 in the multivariate analysis were considered as the independent risk factors for prognosis in the study. In order to avoid the possibility of these biomarkers may have increased the likelihood of achieving chance or spurious results, we performed FDR test by Benjamin Hochberg and ANOVA of repeated measurement data for *P* values of significant immunobiomarkers in multivariate analysis. Odds ratios (ORs) and 95% confidence intervals (CIs) were estimated for each factor. R studio was used to construct the nomogram model of risk assessment using the ‘SvyNom’ and ‘rms’ packages. The box chart combined with scatter chart and line chart were drawn by GraphPad Prism8. SPSS version 25.0 (Chicago, IL, USA) was used for analysis.

## Results

### Clinical Characteristics

**Figure 1:**
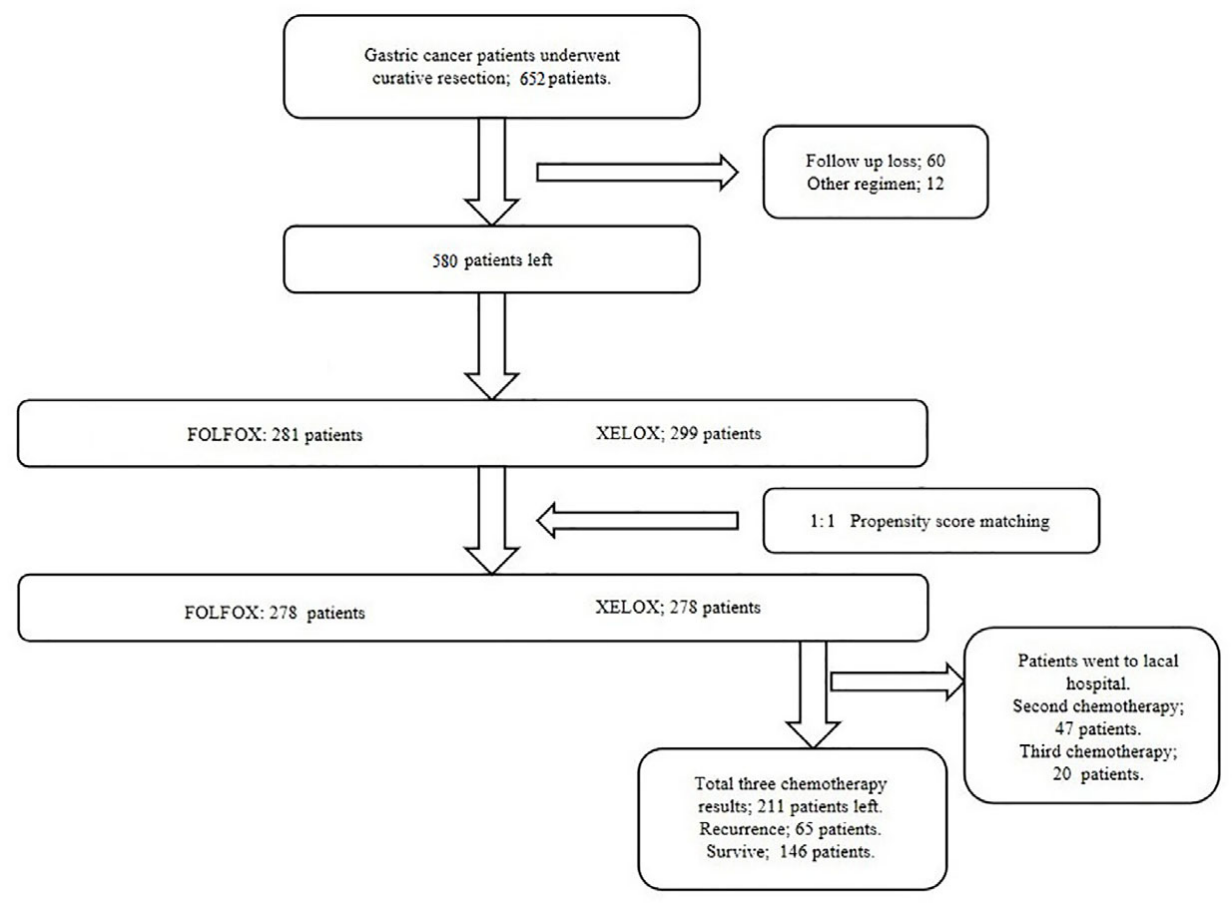
Study protocol designed according to the criteria.

There were 281 and 299 patients in the FOLFOX and XELOX groups, respectively. The median age was 56 years (range: 24–77 years), and the male:female ratio was 425:155. The clinicopathological characteristics of the two groups of patients are summarized in **Table 1**. Before PSM, the two groups of patients had significant differences in vascular invasion (*P* = 0.004). There were 25 (8.9%) and 51 (17.1%) patients with vascular invasion in the FOLFOX and XELOX groups, respectively, and 256 (91.1%) and 248 (82.9%) patients without vascular invasion. After PSM, the two groups were matched 1:1, with 278 patients in each group. Each variable was well balanced without significant difference (All *P* > 0.05) **(** **Table 1**, **Figure 1**).

**Table 1:** Baseline characteristics of patients before and after PSM.

Characteristics	Before PSM			After PSM		
	FOLFOX (281)	XELOX (299)	*p* value	FOLFOX (278)	XELOX (278)	*p* value
Sex			0.986			0.773
Female	75 (26.7)	80 (26.8)		75 (27.0)	72 (25.9)	
Male	206 (73.3)	219 (73.2)		203 (73.0)	206 (74.1)	
Age (years)			0.965			0.712
≤60	195 (69.4)	208 (69.6)		192 (69.1)	196 (70.5)	
>60	86 (30.6)	91 (30.4)		86 (30.9)	82 (29.5)	
Tumor size (mm)			0.440			0.339
≤50	166 (59.1)	186 (62.2)		164 (59.0)	175 (62.9)	
>50	115 (40.9)	113 (37.8)		114 (41.0)	103 (37.1)	
Borrmann type			0.660			0.573
0–2	75 (26.7)	90 (30.1)		75 (27.0)	86 (30.9)	
3	178 (63.3)	181 ((60.5)		176 (63.3)	168 (60.4)	
4	28 (10.0)	28 (9.4)		27 (9.7)	24 (8.7)	
Tumor location			0.866			0.916
Lower third	193 (68.7)	206 (68.9)		190 (68.3)	193 (69.4)	
Middle third	53 (18.9)	52 (17.4)		53 (19.1)	50 (18.0)	
Upper third	28 (10.0)	35 (11.7)		28 (10.1)	30 (10.8)	
Entire stomach	7 (2.4)	6 (2.0)		7 (2.5)	5 (1.8)	
Lymph node dissection			0.489			0.726
D1、 D1+、 D2	42 (14.9)	51 (17.1)		42 (15.1)	45 (16.2)	
D2+	239 (85.1)	248 (82.9)		236 (84.9)	233 (83.8)	
pTNM stage			0.967			0.877
I	26 (9.3)	29 (9.7)		26 (9.4)	29 (10.4)	
II	86 (30.6)	89 (29.8)		86 (30.9)	88 (31.7)	
III	169 (60.1)	181 (60.5)		166 (59.7)	161 (57.9)	
Histological type			0.535			0.858
Well differentiated	98 (34.9)	97 (32.4)		96 (34.5)	94 (33.8)	
Poor differentiation	183 (65.1)	202 (67.6)		182 (65.5)	184 (66.2)	
Vascular infiltration			**0.004**			0.063
Yes	25 (8.9)	51 (17.1)		25 (9.0)	39 (14.0)	
No	256 (91.1)	248 (82.9)		253 (91.0)	239 (86.0)	

Histological type, Borrmann type, lymph node dissection, and pTNM stage were according to the 8th AJCC system. Vascular infiltration was according to the postoperative pathology report. Significant P values are in bold (P < 0.05).

### NLR, PLR, and SII Score

**Figure 2:**
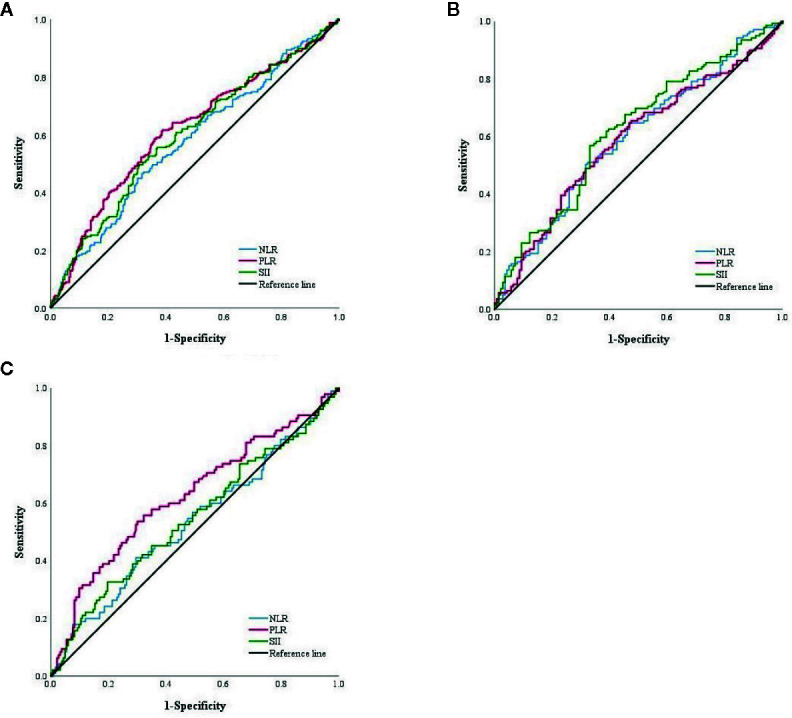
**(A)** ROC curve of NLR, PLR and SII score among total patients in PSM cohort. **(B)** ROC curve of NLR, PLR and SII score of patients with FOLFOX. **(C)** ROC curve of NLR, PLR and SII score of patients with XELOX.

For NLR, PLR and SII score, 2.16, 128.61 and 524.45, respectively, were calculated as the most appropriate cut-off thresholds by the Youden index of the ROC curve for all patients after PSM based on preoperative hematology. The AUC was 0.576 (95% CI: 0.527–0.624), 0.616 (95%CI: 0.568–0.664), and 0.597 (95% CI: 0.549–0.645), respectively (**Figure 2A**). The AUC of NLR, PLR and SII was 0.596 (95% CI: 0.530–0.663), 0.587 (95% CI: 0.520–0.654), and 0.620 (95% CI: 0.554–0.686), respectively, in the FOLFOX group, and 0.533 (95% CI: 0.459–0.606), 0.624 (95% CI: 0.552–0.696), and 0.546 (95% CI: 0.472–0.619) in the XELOX group (**Figures 2B, C**).

### Postoperative Chemotherapy and Patient Survival

**Figure 3:**
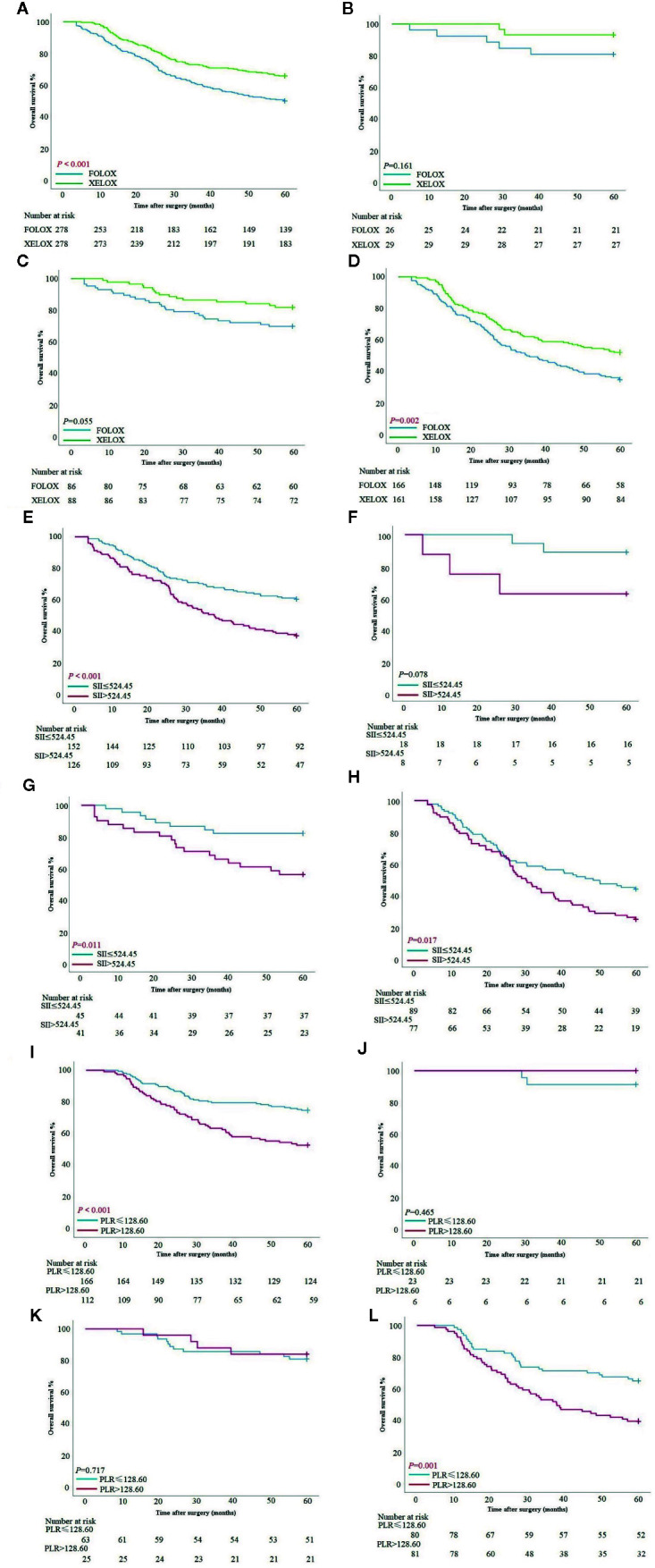
**(A–D)** Survival curves based on patients with FOLFOX and XELOX in all stages, stage I, stage II and III. **(E–H)** Survival curves based on preoperative SII score of patients with FOLFOX in all stage, stage I, stage II and III. **(I–L)** Survival curves based on preoperative PLR of patients with XELOX in all stages, stage I, stage II and III.

In the PSM cohort, the XELOX group had better survival than the FOLFOX group had (*P* < 0.001). In the FOLFOX group, survival time was 59.89 ± 20.70 months, and 5-year survival rate was 50.0%. In the XELOX group, survival time was 60.0 ± 18.13 months, and 5-year survival rate was 65.83%. In stage I and II patients, there was no significant difference in OS between the two groups (*P* = 0.161, *P* = 0.055). In stage III patients, the XELOX group had a better survival rate than the FOLFOX group had (*P* = 0.002). Patients treated with FOLFOX had survival time of 35.15 ± 20.68 months, and 5-year survival rate was 34.94%, and patients treated with XELOX had survival time of 60.0 ± 19.87 months, and 5-year survival rate was 52.17% (**Figures 3A–D**).

The results of the first three postoperative chemotherapy hematological examinations of patients who received XELOX were recorded. Two hundred and seventy-eight patients received the first postoperative chemotherapy regimen, 231 received the second, and 211 received the third. In addition to the first regimen, 67 patients returned to the local hospital for follow-up treatment according to medical advice. Finally, 65 (30.81%) of 211 patients had tumor recurrence (blood metastasis: 29 (44.6%), lymph node metastasis: 15 (23.1%), peritoneal implantation metastasis: 12 (18.5%), recurrence *in situ*: eight (12.3%) and one unknown case). One hundred and forty-six (69.19%) patients survived more than 5 years after treatment.

### Inflammatory Index and Patient Survival

Patients with FOLFOX in the PSM cohort had a significant difference in overall survival between SII >524.45 and SII ≤524.45 [OS: 37.79 ± 21.05 *vs* 60.0 ± 19.68 months, *P <*0.001; hazard ratio (HR): 1.897, 95% CI: 1.355–2.655]. In stage I patients, there was no significant difference between patients with SII >524.45 and SII ≤524.45 (*P* = 0.078). In stage II patients, those with SII >524.45 had worse survival than those with SII ≤524.45 (OS: 60.0 ± 21.12 *vs* 60.0 ± 15.79 months, *P* = 0.011; HR: 2.826, 95% CI: 1.288–60.503). In stage III patients, those with SII >524.45 also had worse survival time (OS: 30.27 ± 19.90 *vs* 48.47 ± 20.97 months, *P* = 0.017; HR: 1.685, 95% CI: 1.144–2.482) (**Figures 3E–H**). SII score had a significant association with PLR, NLR and tumor location at chi-square analysis in clinical and pathological features (*P* < 0.001, *P* < 0.001 and *P* = 0.024) (**Table 2**).

**Table 2:** Chi-square analysis of the connection between inflammation index and clinicopathological features.

Characteristics	FOLFOX			XELOX		
	SII ≤ 524.45	SII>524.45	*P* value	PLR ≤ 128.61	PLR>128.61	*P* value
Sex			0.174			0.265
Female	36 (12.9)	39 (14.0)		39 (14.0)	33 (11.9)	
Male	116 (41.7)	87 (31.3)		127 (45.7)	79 (28.4)	
Age (years)			0.431			0.110
≤60	108 (38.8)	84 (30.2)		123 (44.2)	73 (26.3)	
>60	44 (15.8)	42 (15.1)		43 (15.5)	39 (14.0)	
Tumor size (mm)			0.415			**<0.001**
≤50	93 (33.5)	71 (25.5)		119 (42.8)	56 (20.1)	
>50	59 (21.2)	55 (19.8)		47 (16.9)	56 (20.1)	
SII	–	–	–			**<0.001**
SII ≤ 524.45				154 (55.4)	35 (12.6)	
SII>524.45				12 (4.3)	77 (27.7)	
PLR			**<0.001**	–	–	–
PLR ≤ 128.60	102 (36.7)	19 (6.8)				
PLR>128.60	50 (18.0)	107 (38.5)				
NLR			**<0.001**			**<0.001**
NLR ≤ 2.16	136 (48.9)	28 (10.1)		140 (50.4)	41 (14.7)	
NLR>2.16	16 (5.8)	98 (35.3)		26 (9.4)	71 (25.5)	
Borrmann type			0.351			0.443
0-2	37 (13.3)	38 (13.7)		55 (19.8)	31 (11.2)	
3	102 (36.7)	74 (26.6)		99 (35.6)	69 (24.8)	
4	13 (4.7)	14 (5.0)		12 (4.3)	12 (4.3)	
Tumor location			**0.024**			0.237
Lower third	102 (36.7)	88 (31.7)		117 (42.1)	76 (27.3)	
Middle third	28 (10.1)	25 (9.0)		28 (10.1)	22 (7.9)	
Upper third	21 (7.6)	7 (2.5)		20 (7.2)	10 (3.6)	
Entire stomach	1 (0.4)	6 (2.2)		1 (0.4)	4 (1.4)	
pTNM stage			0.288			**<0.001**
I	18 (6.5)	8 (2.9)		23 (8.3)	6 (2.2)	
II	45 (16.2)	41 (14.7)		63 (22.7)	25 (9.0)	
III	89 (32.0)	77 (27.7)		80 (28.8)	81 (29.1)	
Histological type			0.164			0.770
Well differentiated	47 (16.9)	49 (17.6)		55 (19.8)	39 (14.0)	
Poorly differentiated	105 (37.8)	77 (27.7)		111 (39.9)	73 (26.3)	
Vascular infiltration			0.161			0.131
Yes	17 (6.1)	8 (2.9)		19 (6.8)	20 (7.2)	
No	135 (48.6)	118 (42.4)		147 (52.9)	92 (33.1)	

Histological type, Borrmann type, lymph node dissection, and pTNM stage were according to the 8th AJCC system. Vascular infiltration was according to the postoperative pathology report. Statistically significant P values are in bold (P < 0.05).

Patients treated with XELOX in the PSM cohort had a significant difference in OS between PLR >128.61 and PLR ≤128.61 (OS: 60.0 ± 19.65 *vs* 60.0 ± 16.32 months, *P <* 0.001; HR: 2.178, 95% CI: 1.452–3.266). In stage I and II patients, there was no significant difference in OS between patients with PLR >128.61 and PLR ≤128.61 (*P* = 0.465, *P* = 0.717). In stage III patients, those with PLR >128.6 had worse survival time than those with PLR ≤128.61 (OS: 37.90 ± 20.16 *vs* 60.0 ± 18.57 months, *P* = 0.001; HR: 2.109, 95% CI: 1.324–3.360) (**Figures 3I–L**). PLR had a significant association with tumor size, SII score, NLR and pTNM stage at chi-square analysis in clinical and pathological features (*P* < 0.001, *P* < 0.001, *P* < 0.001, *P* < 0.001) (**Table 2**).

### Univariate and Multivariate Regression Analyses in FOLFOX Groups

To identify the independent risk factors for prognosis of patients with GC in the FOLFOX group, univariate and multivariate analyses based on the Cox risk regression model were implemented. According to univariate analysis, tumor size (*P* < 0.001), SII score (*P* < 0.001), Borrmann type (*P* < 0.001), tumor location (*P* = 0.002) and pTNM stage (*P* < 0.001) were significant. According to multivariate analyses, SII score (*P* = 0.001), Borrmann type (*P* = 0.032) and pTNM stage (*P* < 0.001) were independent risk factors for prognosis (**Table 3**).

**Table 3:** Prognosis factors of patients with GC by univariate and multivariate based on cox regression analysis in FOLFOX group.

Characteristics	FOLFOX			
	Univariate analyses		Multivariate analyses	
	HR (95% CI)	*P* value	HR (95% CI)	*P* value
Sex		0.339	–	–
Male	1			
Female	0.837 (0.581–1.206)			
Age (years)	1.002 (0.984–1.021)	0.817	–	–
Tumor size (mm)	1.013 (1.006–1.019)	**<0.001**	1.001 (0.993–1.009)	0.866
SII	1.001 (1.000–1.001)	**<0.001**	1.001 (1.000–1.001)	**0.001**
Borrmann type		**<0.001**		**0.032**
0-2	1		1	
3	1.526 (0.991–2.348)	0.055	1.303 (0.812–2.092)	0.272
4	4.292 (2.463–7.480)	**<0.001**	2.545 (1.248–5.192)	**0.010**
Tumor location		**0.002**		0.900
Lower third	1		1	
Middle third	1.179 (0.771–1.803)	0.447	1.113 (0.708–1.752)	0.642
Upper third	1.156 (0.669–1.998)	0.603	0.979 (0.538–1.781)	0.945
Entire stomach	4.676 (2.142–10.205)	**<0.001**	1.352 (0.522–3.503)	0.534
pTNM stage		**<0.001**		**<0.001**
I	1		1	
II	1.695 (0.651–4.414)	0.280	1.170 (0.433–3.163)	0.758
III	4.716 (1.923–11.571)	**0.001**	3.091 (1.194–8.002)	**0.020**
Histological type		0.777	–	–
Well differentiated	1			
Poorly differentiated	1.052 (0.739–1.498)			
Vascular infiltration		0.062	–	–
No	1			
Yes	1.642 (0.975–2.766)			

Histological type, Borrmann type, lymph node dissection, and pTNM stage were according to the 8th AJCC system. Vascular infiltration was according to the postoperative pathology report. Statistically significant P values are in bold (P < 0.05).

### Univariate and Multivariate Regression Analyses in XELOX Groups

**Figure 4:**
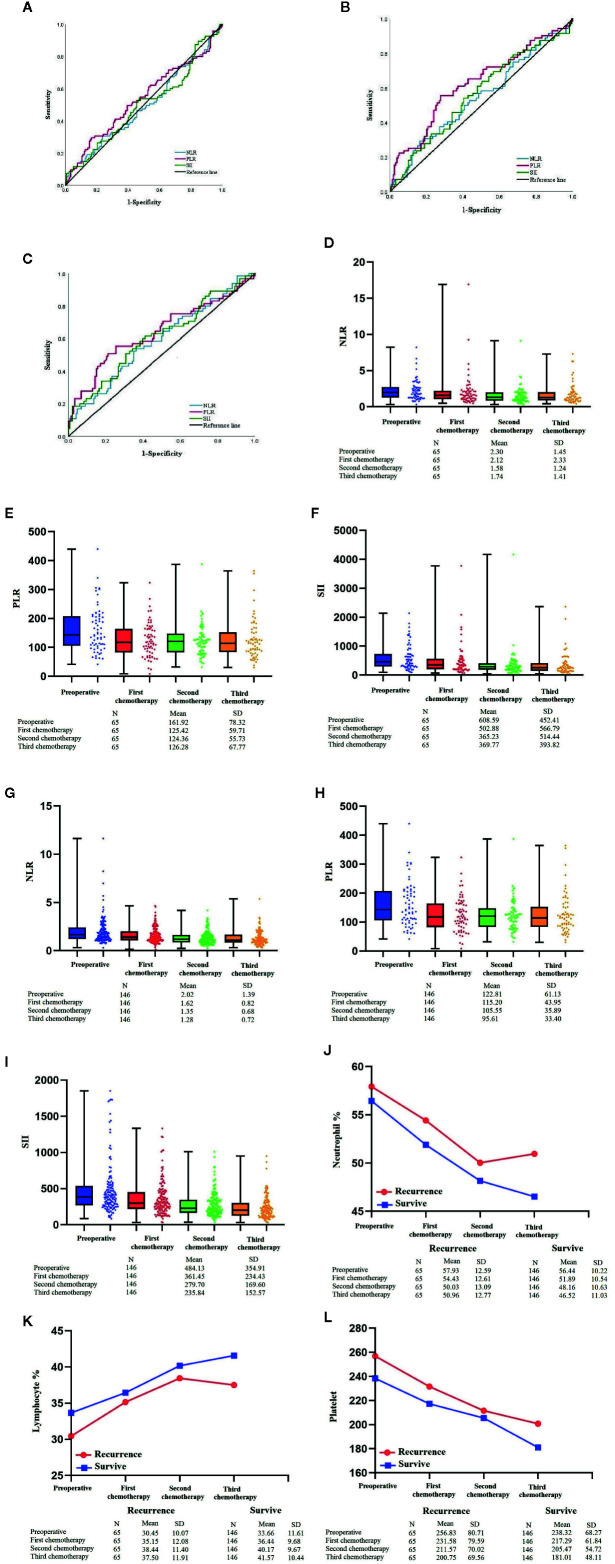
**(A–C)** ROC curve of PLR of patients with XELOX from first to third postoperative chemotherapy regimens. **(D–F)** Box plot combined with scatter plot of NLR, PLR and SII score of patients with tumor recurrence from preoperative period to the third postoperative chemotherapy regimen. **(G–I)** Box plot combined with scatter plot of NLR, PLR and SII score of patients who survived >5 years from preoperative period to the third postoperative chemotherapy regimen. **(J–L)** Line chart of lymphocytes, neutrophils and platelets of patients with XELOX from preoperative period to the third postoperative chemotherapy regimen.

Previous results showed that the OS of patients in the XELOX group was significantly better than in the FOLFOX group, which mainly in those with stage III. We performed detailed statistical analysis on the first three chemotherapy regimens of patients in the XELOX group. According to hematological examination of the patients in the first three postoperative chemotherapy regimens, through the ROC curve, AUC of NLR was 0.494, 0.547 and 0.590, compared with 0.538, 0.628 and 0.641 in PLR and 0.498, 0.565 and 0.609 in SII score, respectively (**Figures 4A–C**).

Univariate and multivariate analysis based on Cox risk regression model were performed according to clinical and pathological factors, including sex, age, tumor diameter, PLR, Borrmann type, tumor location, pTNM stage, histological type and vascular infiltration from the preoperative period to the second postoperative chemotherapy regimen. The analysis of preoperative and each time of postoperative chemotherapy were showed in **Supplement 1**. For patients with second time of chemotherapy after radical gastrectomy, tumor size (*P* = 0.008), PLR (*P* = 0.014) and pTNM stage (*P* = 0.009) were independent risk factors for DFS (**Table 4-A**). Tumor size (*P* = 0.009), PLR (*P* = 0.011) and pTNM stage (*P* = 0.008) were independent risk factors for OS (**Table 4-B**). In order to avoid the possibility that PLR may have increased the likelihood of achieving chance or spurious results, we performed FDR test and ANOVA of repeated measurement data for *P* values in multivariate analysis. In addition, the PLR values of 211 patients of each treatment were shown by Line Chart (**Supplement 2** and **3**).

**Table 4A:** Prognosis factors for DFS of patients with GC by multivariate based on cox regression analysis in XELOX group.

Characteristics	XELOX Second postoperative chemotherapy (Patients=231)			
	Univariate analyses		Multivariate analyses	
	HR (95% CI)	*P* value	HR (95% CI)	*P* value
Sex		0.367	–	–
Male	1			
Female	1.268 (0.757–2.123)			
Age (years)	1.017 (0.991–1.044)	0.198	–	–
Tumor size (mm)	1.020 (1.012–1.027)	**<0.001**	1.014 (1.004–1.024)	**0.008**
PLR	1.009 (1.004–1.013)	**<0.001**	1.006 (1.001–1.012)	**0.014**
Borrmann type		**<0.001**		0.286
0-2	1		1	
3	2.583 (1.341–4.976)	**0.005**	1.741 (0.876–3.462)	0.114
4	5.414 (2.420–12.113)	**<0.001**	1.679 (0.633–4.449)	0.297
Tumor location		**0.023**		0.165
Lower third	1		1	
Middle third	1.055 (0.569–1.956)	0.865	0.812 (0.433–1.525)	0.518
Upper third	1.658 (0.835–3.291)	0.149	1.969 (0.968–4.002)	0.061
Entire stomach	4.446 (1.593–12.412)	**0.004**	1.467 (0.423–5.086)	0.546
pTNM stage		**<0.001**		**0.009**
I	1		1	
II	4.494 (0.588–34.351)	0.148	3.369 (0.437–25.984)	0.244
III	13.666 (1.892–98.689)	**0.010**	7.615 (1.029–56.334)	**0.047**
Histological type		0.636	–	–
Well differentiated	1			
Poorly differentiated	1.129 (0.684–1.864)			
Vascular infiltration		0.487	–	–
No	1			
Yes	1.237 (0.679–2.256)			

Histological type, Borrmann type, lymph node dissection, and pTNM stage were according to the 8th AJCC system. Vascular infiltration was according to the postoperative pathology report. Statistically significant P values are in bold (P < 0.05).

**Table 4B:** Prognosis factors for OS of patients with GC by multivariate based on cox regression analysis in XELOX group.

Characteristics	XELOX Second postoperative chemotherapy (Patients = 231)			
	Univariate analyses		Multivariate analyses	
	HR (95% CI)	*P* value	HR (95% CI)	*P* value
Sex		0.357	–	–
Male	1			
Female	1.274 (0.761–2.134)			
Age (years)	1.018 (0.991–1.044)	0.192	–	–
Tumor size (mm)	1.019 (1.012–1.026)	**<0.001**	**1.013 (1.003**–**1.022)**	**0.009**
PLR	1.009 (1.005–1.014)	**<0.001**	**1.007 (1.002**–**1.012)**	**0.011**
Borrmann type		**<0.001**		0.250
0-2	1		1	
3	2.619 (1.360–5.044)	0.004	1.785 (0.899–3.544)	0.098
4	5.517 (2.466–12.342)	**<0.001**	1.807 (0.685–4.769)	0.232
Tumor location		**0.023**		0.170
Lower third	1		1	
Middle third	1.040 (0.561–1.927)	0.902	0.813 (0.434–1.523)	0.518
Upper third	1.681 (0.846–3.338)	0.138	1.952 (0.962–3.962)	0.064
Entire stomach	4.399 (1.575–12.285)	**0.005**	1.505 (0.438–5.179)	0.516
pTNM stage		**<0.001**		**0.008**
I	1		1	
II	4.495 (0.588–34.361)	0.148	3.297 (0.427–25.486)	0.253
III	13.615 (1.885–98.323)	0.010	**7.614 (1.029**–**56.343)**	**0.047**
Histological type		0.608	–	–
Well differentiated	1			
Poorly differentiated	1.140 (0.691–1.882)			
Vascular infiltration		0.468	–	–
No	1			
Yes	1.249 (0.685–2.277)			

Histological type, Borrmann type, lymph node dissection, and pTNM stage were according to the 8th AJCC system. Vascular infiltration was according to the postoperative pathology report. FDR test by Benjamin Hochberg and ANOVA of repeated measurement data were performed for P values of significant immunobiomarkers in multivariate analysis. Statistically significant P values are in bold (P < 0.05).

### Dynamic Changes of Inflammation Index and Circulating Immune Cells

For 65 patients with tumor recurrence after the third postoperative chemotherapy regimen, the NLRs were 2.30 ± 1.45 (mean ± standard deviation), 2.12 ± 2.33, 1.58 ± 1.24 and 1.74 ± 1.41; PLRs were 161.92 ± 78.32, 125.42 ± 59.71, 124.36 ± 55.73 and 126.28 ± 67.77, and SII scores were 608.59 ± 452.41, 502.88 ± 566.79, 365.23 ± 514.44 and 369.77 ± 393.82 (**Figures 4D–F**). For circulating immune cells, the percentages of neutrophils were 57.93 ± 12.59, 54.43 ± 12.61, 50.03 ± 13.09 and 50.96 ± 12.77; the percentages of lymphocytes were 30.45 ± 10.07, 35.15 ± 12.08, 38.44 ± 11.40 and 37.50 ± 11.91, and platelet count was 256.83 ± 80.71, 231.58 ± 79.59, 211.57 ± 70.02 and 200.75 ± 69.56 (**Figures 4J–L**).

For 146 patients who survived for >5 years from the preoperative period to the third postoperative chemotherapy regimen, the NLRs were 2.02 ± 1.39, 1.62 ± 0.82, 1.35 ± 0.68 and 1.28 ± 0.72; PLRs were 122.81 ± 61.13, 115.20 ± 43.95, 105.55 ± 35.89 and 95.61 ± 33.40; and SII scores were 484.13 ± 354.91, 361.45 ± 234.43, 279.70 ± 169.60 and 235.84 ± 152.57 (**Figures 4G–I**). For circulating immune cells, the percentages of neutrophils were 56.44 ± 10.22, 51.89 ± 10.54, 48.16 ± 10.63 and 46.52 ± 11.03; the percentages of lymphocytes were 33.66 ± 11.61, 36.44 ± 9.68, 40.17 ± 9.67 and 41.57 ± 10.44; and platelet count was 238.32 ± 68.27, 217.29 ± 61.84, 205.47 ± 54.72 and 181.01 ± 48.11 (**Figures 4J–L**).

### Nomogram in XELOX Group

**Figure 5:**
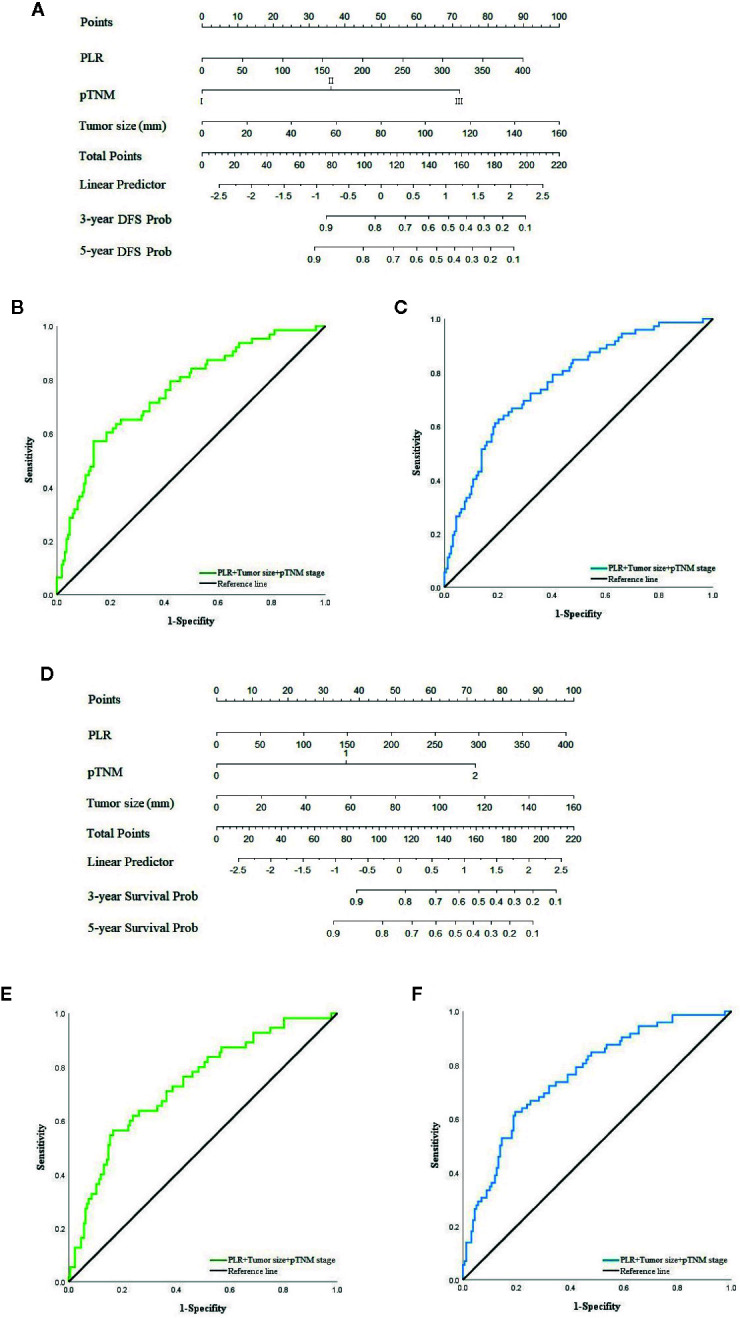
**(A)** Nomogram model predicting 3 and 5 years recurrence of patients in XELOX group. **(B)** ROC curve of nomogram model predicting 3-year recurrence. **(C)** ROC curve of nomogram model predicting 5-year recurrence. **(D)** Nomogram model predicting 3 and 5 years survival of patients in XELOX group. **(E)** ROC curve of nomogram model predicting 3-year survival. **(F)** ROC curve of nomogram model predicting 5-year survival.

In the second chemotherapy regimen, due to the univariate and multivariate regression analyses, PLR, tumor size and pTNM stage were independent risk factors that significantly correlated with the recurrence and prognosis of patients in the XELOX group, based on the Cox risk regression model. We combined these clinical features with DFS and OS to construct nomogram models to predict the recurrence and prognosis of patients (**Figures 5A, D**). The AUC of the model that predicted recurrence within 3 and 5 years was 0.757 (95% CI: 0.687–827) **(** **Figure 5B**) and 0.765 (95% CI: 0.699–0.830) (**Figure 5C**), respectively. The sensitivity was 57.1% and 62.5%, respectively, and the specificity was 86.3% and 79.9%, respectively. The AUC of the model that predicted prognosis within 3 and 5 years was 0.735 (95% CI: 0.659–0.810) (**Figure 5E**) and 0.763 (95% CI: 0.698–0.828) (**Figure 5F**), respectively. The sensitivity was 56.4 and 62.5%, respectively, and the specificity was 83.5 and 80.5%, respectively.

## Discussion

Radical gastrectomy is still the standard treatment for GC. To inhibit metastasis of tumor cells, adjuvant treatments are becoming abundant, such as neoadjuvant chemotherapy, postoperative chemotherapy, immunotherapy, and targeted therapy. However, there are still significant variations worldwide in treatment outcome because of the high heterogeneity of GC. For example, patients in Asia mainly have distal, intestinal and HER-2-positive GC, while those in western countries are mainly proximal, diffuse and HER-2-negative GC, and intestinal GC is more sensitive to postoperative chemotherapy, especially oxaliplatin-based chemotherapy (15, 16). Besides, on the timing of chemotherapy, the MAGIC trial confirmed that preoperative neoadjuvant chemotherapy can increase the 5-year survival rate of stage II–III GC patients from 23 to 36%, which makes neoadjuvant chemotherapy widely used in Europe. In Asia, neoadjuvant chemotherapy also has been shown to be effective in improving surgical resection rates (17, 18), but postoperative chemotherapy has been widely shown to have good clinical efficacy for decades, which, combined with radical gastrectomy, has become the standard treatment mode. Therefore, to select suitable chemotherapy regimens for Chinese patients, we compared the long-term efficacy between FOLFOX and XELOX. We found that OS of patients receiving XELOX was significantly better than those receiving FOLFOX, and this difference was mainly found in patients with stage III GC, which is the same as the previous study by Kabsoo et al. study on XELOX (19).

The CLASSIC trial confirmed that XELOX chemotherapy increased the 5-year survival rate of patients by 9% and reduced the incidence of chemotherapy toxicity to 10% (5, 6). Park et al. (20) also found that for advanced GC patients who did not receive any treatment, their overall remission rate after treatment with XELOX regimen was 63%, and OS was extended to 11.9 months. These trials showed satisfactory clinical results for XELOX treatment for GC. The sensitivity of cancer cells to drug treatment depends not only on sufficient drugs reaching the target cells, but also on the drug sensitivity of the tumor cells (21, 22). Capecitabine is a precursor of fluorouracil and has no anticancer effect itself, but has cytotoxicity at the location of the liver and solid tumors, thereby increasing the drug concentration in tumor cells, while minimizing the systemic toxicity of chemotherapy. Since it was first approved for the treatment of metastatic colon cancer in 2001, it has been used frequently in the gastrointestinal tract because of its simple administration route and therapeutic effectiveness (23). The third-generation antitumor platinum drug oxaliplatin ((1R, 2R-diamminocyclohexane) oxalatoplatinum (II)) has a different antitumor effect compared with conventional cisplatin. The hydrated derivatives it forms act on DNA structure and mainly form interchain crosslinks to bend and unwind DNA. Meanwhile, the affinity of high-mobility group proteins for oxaliplatin interchain crosslinks is significantly lower than that of cisplatin, which makes the antitumor activity of oxaliplatin significantly stronger than that of cisplatin (24–26). Besides, it shows a good clinical effect on HER-2-positive and intestinal GC, mainly in China and Japan. Tumor cells of intestinal GC are rich in ribosomes and lack lysosomes and mucus, which makes them more responsive to oxaliplatin chemotherapy than diffuse GC cells (27, 28). Additionally, due to the lower intake of folic acid in Asian patients and the significant difference in *CYP2A6* gene between Asian and Caucasian populations, Asian patients are more tolerant of treatment-related toxicity of chemotherapeutic drugs (29). However, Nozomu Fuse et al. (30) found that the incidence of chemotherapy-related toxicity in Japanese patients treated with XELOX increased significantly to 94%, although they suspected that it was due to the large number of older patients in the study. The clinical effect of XELOX should be verified by further, extensive clinical data. In our study, 146 patients (69.2%) who received XELOX treatment achieved 5-year survival, which was significantly higher than the clinically common 50% response rate (31), and no serious chemotherapy toxicity response occurred. Although this result might be related to the fact that only the first three chemotherapy results were recorded in this study, it confirmed the CLASS study and indicated that XELOX regimen is suitable for postoperative treatment of Chinese GC patients.

Prognosis of patients with resectable GC is based on histopathological criteria of tumor invasion according to the Union for International Cancer Control and AJCC TNM classification system, which could supply useful but incomplete prognostic information. On the other hand, the connection between tumor immunology and prognosis has been gradually recognized, which is also considered as a potential biomarker and guidance for appropriate treatment (32–35). *New England Journal of Medicine* has reported that for patients with invasive and indolent lymphoma, rituximab combined with macrophage checkpoint inhibitor 5F9 has shown good results in clinical treatment (36). However, histopathological observation of the immune response of the tumor microenvironment is limited by the randomness of selection of tissue sites, and the inflammation indexes in peripheral blood have been verified to evaluate the prognosis of patients with esophageal cancer, renal cancer and GC. However, these indexes cannot reflect the individual patient’s immune status like the former. Numerous of evidence indicates that systemic inflammatory response is related to the effect of chemotherapy (37). Huang et al. (38) found that patients with a combined Neutrophil/platelet/lymphocyte/differentiation Score (CNPLDS) of six to nine are less sensitive to first-line chemotherapeutic drugs postoperatively than those with a score of one to five. We found that SII score was an independent risk factor for prognosis in the FOLFOX group and PLR in the second time of XELOX postoperative chemotherapy regimen was an independent predictor using the Cox risk regression model, which could evaluate the clinical efficacy of the corresponding treatment. We also found that high PLR had a significant association with tumor size and pTNM stage through the chi-square test, which was related to deeper tumor invasion, and presence of not only local lymph node metastasis, but also a distant metastasis. Fridman et al. (39) also demonstrated that the immune system plays an important role in metastasis. Such progress in the XELOX group in our study was reflected by dynamic changes in PLR and corresponding immune cells.

Postoperative PLR could assess the sensitivity to XELOX of patients with GC, which also indicated that platelet and lymphocytes played important roles during treatment. Additionally, we found that in patients with tumor recurrence, inflammation biomarkers NLR, PLR and SII score were increased during the second to third chemotherapy regimens. Among the related circulating immune cells, there an increase in neutrophil percentage as well as a decline in lymphocyte percentage, and the decline in platelet count was lower than that of long-term surviving patients. By contrast, for 146 long-term surviving patients, inflammation biomarkers NLR, PLR and SII score, neutrophil percentage and platelet count showed a downward trend, but lymphocyte percentage showed a continuous increase, which indicated that not only the postoperative PLR, but also the dynamic changes in PLR during treatment could evaluate the sensitivity of patients to XELOX chemotherapy. This might be related to the 29 patients (44.6%) with blood metastasis among the patients with GC recurrence, which was higher than 34.2% in the study by Yoo et al. (40). Although the above dynamic changes did not show significant differences, this trend was worthy of further study. It is known that in the circulating immune system, lymphocytes kill tumor cells and inhibit distant metastasis, and a large increase in neutrophils can secrete cytokines interleukin (IL)-1, IL-10, interferon-γ and tumor necrosis factor-α and other factors to inhibit lymphocytes (CD4^+^ and CD8^+^ cells) and natural killer cells, promote tumor immune escape, and enhance tumor cell resistance. The inflammatory reaction around neutrophils triggers a wide cascade effect that causes damage to contact tissues and nonspecific inflammatory response, and promotes tumor cell implantation and recurrence (41–44). Platinum-based chemotherapeutic drugs destroy vascular endothelial cells to produce Von Willebrand factor, while circulating platelet can promote angiogenesis *via* this factor. Additionally, platelet also promote tumor metastasis and angiogenesis by releasing various growth factors such as vascular endothelial growth factor-A. The platelet formed can also promote tumor cell immune escape and resistance to chemotherapeutic drugs. The progression, metastasis and recurrence of tumors lead to changes in systemic inflammatory response, which can be indirectly manifested by continuous hematological testing (45–47). For patients with abnormal inflammation indexes and immune cells during the second to third chemotherapy regimens, whether they can be remedied by adding cetuximab (48) or changing the regimen will be the direction of our next study.

Although studies that have focused on predicting prognosis of patients or guiding treatment by inflammatory index have been widely used, it is difficult to individualize evaluation. With the development of big data for cancer research and real world studies, nomograms combining clinicopathological features and inflammatory indexes to evaluate the prognosis of GC have been widely used clinically. Liu et al. (49) found that a nomogram that combined systemic prognostic score, tumor location and TNM stage can predict the prognosis of stage II–III GC with postoperative chemotherapy. We analyzed the clinical significance of PLR through the Gastric Cancer Information Management System v1.2 of Harbin Medical University Cancer Hospital database. According to multivariate analysis, PLR of the second postoperative chemotherapy regimen, tumor size and pTNM stage were independent factors that significantly correlated with recurrence and prognosis of patients in the XELOX group. A nomogram model that combined the above factors was constructed to predict the recurrence and prognosis for 3 and 5 years. Through ROC curve analysis, it was found that AUC of the nomogram that predicted the recurrence of patients for 3 and 5 years were 0.757 and 0.765, sensitivity was 57.1 and 62.5%, and specificity was 86.3 and 79.9%, respectively. The AUC of the nomogram that predicted the prognosis of patients for 3 and 5 years were 0.735 and 0.763, sensitivity was 56.4 and 62.5%, and specificity was 83.5 and 80.5%, respectively. The prediction model established by PLR, tumor size and pTNM merits further clinical verification and application.

## Limitations

This retrospective study still had some limitations. First, although PSM was used to deal with the bias between groups, there may still be potential factors that affected the results. Second, this study was mainly aimed at inflammation indexes of Chinese GC patients who receive chemotherapy, thus, whether the results are applicable to other populations needs verification. Finally, further analysis is needed to evaluate the efficacy of FOLFOX chemotherapy by peripheral blood immune biomarkers.

## Conclusion

The survival was superior in the XELOX over the FOLFOX group, although this is the case overall, statistical significance was only reached in those with stage III disease, not those in stage I and stage II disease (*P* = 0.161 and 0.055). Also, in the XELOX group, the PLR predicted for prognosis in the stage II and III patients only, not the stage I patients (*P* = 0.078). Preoperative SII score was an independent risk factor for prognosis in the FOLFOX group, while PLR of the second postoperative chemotherapy regimen was an independent risk factor for prognosis in the XELOX group. The nomogram constructed by PLR, tumor size and pTNM stage can evaluate the recurrence and prognosis of patients who receive XELOX.

## Data Availability Statement

Publicly available datasets were analyzed in this study. This data can be found here: The Gastric Cancer Information Management System v1.2 of Harbin Medical University Cancer Hospital (Copyright No.2013SR087424, http://www.sgihmu.com).

## Ethics Statement

The studies involving human participants were reviewed and approved by Harbin Medical University Cancer Hospital Ethics Committee of (Approval Number : SHGC-1029). The patients/participants provided their written informed consent to participate in this study.

## Author Contributions

XY and TF designed and conceived this project; they contributed equally to this work. XY, TF, and YiW interpreted and analyzed the data. YX revised the manuscript for important intellectual content; XY, TF, YiW, CL, YuW, and DZ participated in the patient information collection. All authors contributed to the article and approved the submitted version.

## Funding

This work was supported by Nn10 program of Harbin Medical University Cancer Hospital, China (No. Nn10 PY 2017-03). Approved by Harbin Medical University Cancer Hospital.

## Conflict of Interest

The authors declare that the research was conducted in the absence of any commercial or financial relationships that could be construed as a potential conflict of interest.
