# Supplementary material for: Efficacy of Postoperative FOLFOX Versus XELOX Chemotherapy for Gastric Cancer and Prognostic Value of Platelet–Lymphocyte Ratio in Patients Receiving XELOX
Source: Front Oncol. 2020 Dec 23;10:584772. doi: 10.3389/fonc.2020.584772 (PMC7786002; doi:10.3389/fonc.2020.584772)

**Supplement 1**

Prognosis factors of patients with GC by multivariate based on cox regression analysis in XELOX group from preoperative period to second postoperative chemotherapy regimen.

| **Characteristics** | **XELOX Preoperative (Patients=278)** | | | | |
| --- | --- | --- | --- | --- | --- |
|  | **Univariate analyses** | | **Multivariate analyses** | | |
|  | **HR (95% CI)** | ***P* value** | **HR (95% CI)** | ***P* value** | **Q value** |
| Sex |  | 0.655 | - | - | - |
| Male | 1 |  |  |  |  |
| Female | 1.108 (0.706-1.740) |  |  |  |  |
| Age (years) | 1.017 (0.994-1.041) | 0.149 | - | - | - |
| Tumor size (mm) | 1.016 (1.010-1.023) | **＜0.001** | 1.006 (0.997-1.015) | 0.194 | - |
| PLR | 1.004 (1.001-1.006) | **0.002** | 1.002 (1.000-1.005) | 0.095 | - |
| Borrmann type |  | **＜0.001** |  | 0.056 |  |
| 0-2 | 1 |  | 1 |  |  |
| 3 | 2.525 (1.439-4.433) | **0.001** | 1.739 (0.966-3.131) | 0.065 |  |
| 4 | 5.498 (2.712-11.146) | **＜0.001** | 2.762 (1.181-6.458) | **0.019** | **0.027** |
| Tumor location |  | 0.077 |  | 0.545 | - |
| Lower third | 1 |  | 1 |  |  |
| Middle third | 1.144 (0.676-1.936) | 0.616 | 0.891 (0.522-1.520) | 0.671 |  |
| Upper third | 1.319 (0.710-2.451) | 0.381 | 1.463 (0.774-2.766) | 0.242 |  |
| Entire stomach | 3.730 (1.352-10.287) | **0.011** | 1.370 (0.427-4.392) | 0.596 |  |
| pTNM stage |  | **＜0.001** |  | **＜0.001** | **＜0.010** |
| Ⅰ | 1 |  | 1 |  |  |
| Ⅱ | 2.830 (0.651-12.306) | 0.165 | 2.334 (0.532-10.245) | 0.261 |  |
| Ⅲ | 9.208 (2.261-37.495) | **0.002** | 6.132 (1.475-25.483) | **0.013** | **0.022** |
| Histological type |  | 0.524 | - | - | - |
| Well differentiated | 1 |  |  |  |  |
| Poor differentiated | 1.151 (0.747-1.774) |  |  |  |  |
| Vascular infiltration |  | 0.437 | - | - | - |
| No | 1 |  |  |  |  |
| Yes | 1.245 (0.717-2.161) |  |  |  |  |
| **Characteristics** | **XELOX First postoperative chemotherapy (Patients=278)** | | | | |
|  | **Univariate analyses** | | **Multivariate analyses** | | |
|  | **HR (95% CI)** | ***P* value** | **HR (95% CI)** | ***P* value** | **Q value** |
| Sex |  | 0.655 | - | - | - |
| Male | 1 |  |  |  |  |
| Female | 1.108 (0.706-1.740) |  |  |  |  |
| Age (years) | 1.017 (0.994-1.041) | 0.149 | - | - | - |
| Tumor size (mm) | 1.016 (1.010-1.023) | **＜0.001** | 1.008 (0.999-1.017) | 0.089 | - |
| PLR | 1.000 (0.999-1.001) | 0.793 | - | - | - |
| Borrmann type |  | **＜0.001** |  | 0.079 |  |
| 0-2 | 1 |  | 1 |  |  |
| 3 | 2.525 (1.439-4.433) | **0.001** | 1.775 (0.989-3.185) | 0.054 |  |
| 4 | 5.498 (2.712-11.146) | **＜0.001** | 2.466 (1.070-5.683) | **0.034** | **0.043** |
| Tumor location |  | 0.077 |  | 0.638 | - |
| Lower third | 1 |  | 1 |  |  |
| Middle third | 1.144 (0.676-1.936) | 0.616 | 0.902 (0.529-1.538) | 0.704 |  |
| Upper third | 1.319 (0.710-2.451) | 0.381 | 1.404 (0.745-2.647) | 0.294 |  |
| Entire stomach | 3.730 (1.352-10.287) | **0.011** | 1.300 (0.405-4.170) | 0.659 |  |
| pTNM stage |  | **＜0.001** |  | **＜0.001** | **＜0.010** |
| Ⅰ | 1 |  | 1 |  |  |
| Ⅱ | 2.830 (0.651-12.306) | 0.165 | 2.415 (0.551-10.581) | 0.242 |  |
| Ⅲ | 9.208 (2.261-37.495) | **0.002** | 6.635 (1.604-27.440) | **0.009** | **0.030** |
| Histological type |  | 0.524 | - | - | - |
| Well differentiated | 1 |  |  |  |  |
| Poor differentiated | 1.151 (0.747-1.774) |  |  |  |  |
| Vascular infiltration |  | 0.437 | - | - | - |
| No | 1 |  |  |  |  |
| Yes | 1.245 (0.717-2.161) |  |  |  |  |
| **Characteristics** | **XELOX Second postoperative chemotherapy (Patients=231)** | | | | |
|  | **Univariate analyses** | | **Multivariate analyses** | | |
|  | **HR (95% CI)** | ***P* value** | **HR (95% CI)** | ***P* value** | **Q value** |
| Sex |  | 0.357 | - | - | - |
| Male | 1 |  |  |  |  |
| Female | 1.274 (0.761-2.134) |  |  |  |  |
| Age (years) | 1.018 (0.991-1.044) | 0.192 | - | - | - |
| Tumor size (mm) | 1.019 (1.012-1.026) | **＜0.001** | **1.013 (1.003-1.022)** | **0.009** | **0.023** |
| PLR | 1.009 (1.005-1.014) | **＜0.001** | **1.007 (1.002-1.012)** | **0.011** | **0.022** |
| Borrmann type |  | **＜0.001** |  | 0.250 | - |
| 0-2 | 1 |  | 1 |  |  |
| 3 | 2.619 (1.360-5.044) | **0.004** | 1.785 (0.899-3.544) | 0.098 |  |
| 4 | 5.517 (2.466-12.342) | **＜0.001** | 1.807 (0.685-4.769) | 0.232 |  |
| Tumor location |  | **0.023** |  | 0.170 | - |
| Lower third | 1 |  | 1 |  |  |
| Middle third | 1.040 (0.561-1.927) | 0.902 | 0.813 (0.434-1.523) | 0.518 |  |
| Upper third | 1.681 (0.846-3.338) | 0.138 | 1.952 (0.962-3.962) | 0.064 |  |
| Entire stomach | 4.399 (1.575-12.285) | **0.005** | 1.505 (0.438-5.179) | 0.516 |  |
| pTNM stage |  | **＜0.001** |  | **0.008** | **0.040** |
| Ⅰ | 1 |  | 1 |  |  |
| Ⅱ | 4.495 (0.588-34.361) | 0.148 | 3.297 (0.427-25.486) | 0.253 |  |
| Ⅲ | 13.615 (1.885-98.323) | **0.010** | **7.614 (1.029-56.343)** | **0.047** | 0.052 |
| Histological type |  | 0.608 | - | - | - |
| Well differentiated | 1 |  |  |  |  |
| Poor differentiated | 1.140 (0.691-1.882) |  |  |  |  |
| Vascular infiltration |  | 0.468 | - | - | - |
| No | 1 |  |  |  |  |
| Yes | 1.249 (0.685-2.277) |  |  |  |  |

Histological type, Borrmann type, lymph node dissection, and pTNM stage were according to the 8th AJCC system. Vascular infiltration was according to the postoperative pathology report. Statistically significant *P* values are in bold (*P*<0.05).

**Supplement 2**

| **Groups** | **Time** | **Value** | **F value** | ***P* value** |
| --- | --- | --- | --- | --- |
| Recurrence patients (n=65) | Preoperative | 161.92±78.32 | 16.961 | ＜0.001 |
|  | First postoperative chemotherapy | 125.42±59.71 |  |  |
|  | Second postoperative chemotherapy | 124.36±55.73 |  |  |
|  | Third postoperative chemotherapy | 126.28±67.77 |  |  |
| Survival patients (n=146) | Preoperative | 122.81±61.13 |  |  |
|  | First postoperative chemotherapy | 115.20±43.95 |  |  |
|  | Second postoperative chemotherapy | 105.55±35.89 |  |  |
|  | Third postoperative chemotherapy | 95.61±33.40 |  |  |

**Supplement 3**


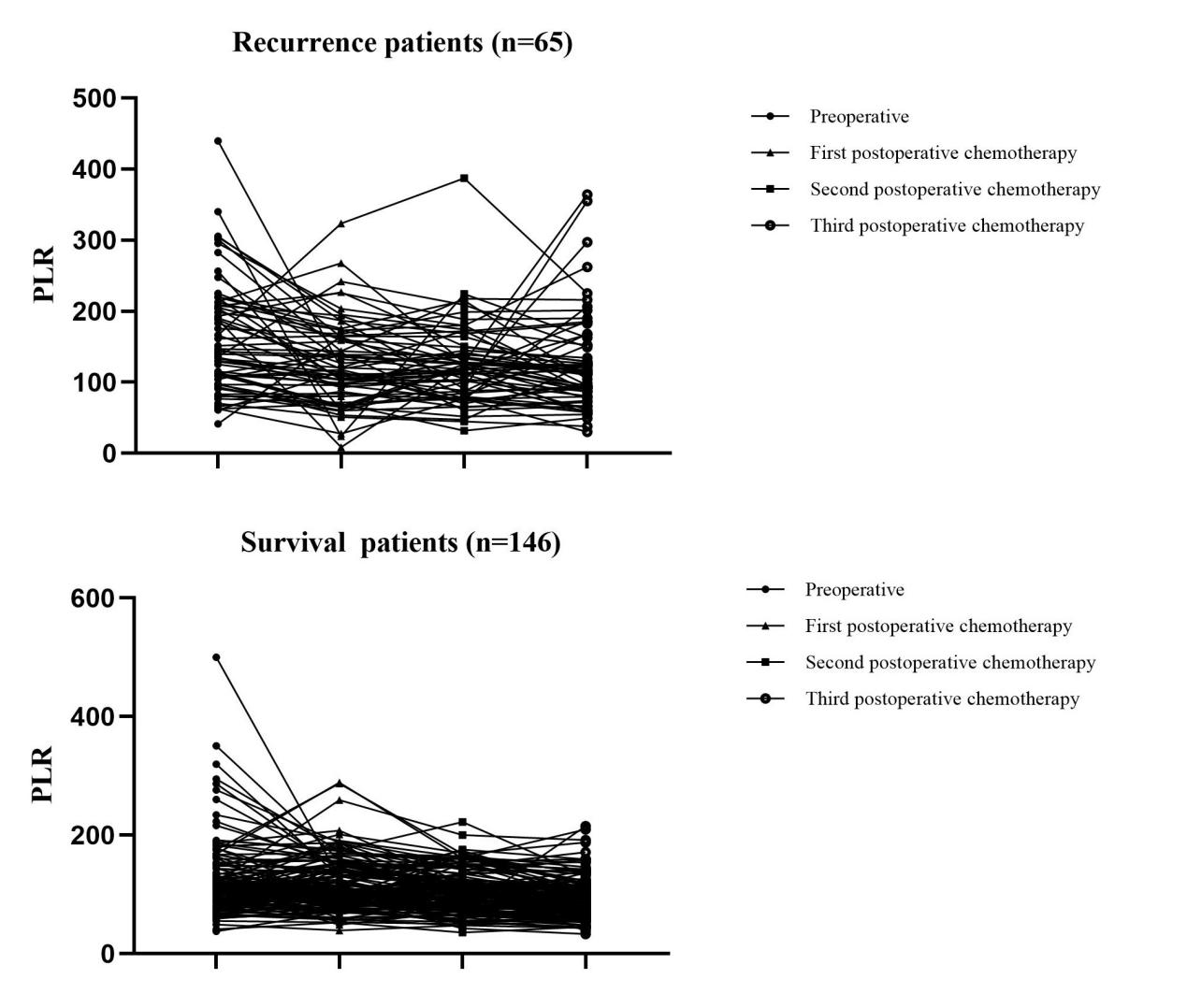

Supplement: Supplementary file 1 [file Table_1.docx]
